# Health-related quality of life in patients with ANCA vasculitides compared to rheumatoid arthritis: a cross-sectional comparative study

**DOI:** 10.1093/rheumatology/kead214

**Published:** 2023-05-12

**Authors:** Alexandros Panagiotopoulos, Konstantinos Thomas, Evangelia Argyriou, Aglaia Chalkia, Noemin Kapsala, Christos Koutsianas, Evgenia Mavrea, Dimitrios Petras, Dimitrios T Boumpas, Dimitrios Vassilopoulos

**Affiliations:** Joint Rheumatology Program, Clinical Immunology-Rheumatology Unit, 2nd Department of Medicine and Laboratory, National and Kapodistrian University of Athens School of Medicine, General Hospital of Athens Hippokration, Athens, Greece; Joint Rheumatology Program, Clinical Immunology-Rheumatology Unit, 4th Department of Medicine, National and Kapodistrian University of Athens School of Medicine, Attikon General Hospital, Athens, Greece; Rheumatology Unit, Sismanoglio General Hospital, Athens, Greece; Nephrology Department, General Hospital of Athens Hippokration, Athens, Greece; Joint Rheumatology Program, Clinical Immunology-Rheumatology Unit, 4th Department of Medicine, National and Kapodistrian University of Athens School of Medicine, Attikon General Hospital, Athens, Greece; Joint Rheumatology Program, Clinical Immunology-Rheumatology Unit, 2nd Department of Medicine and Laboratory, National and Kapodistrian University of Athens School of Medicine, General Hospital of Athens Hippokration, Athens, Greece; Joint Rheumatology Program, Clinical Immunology-Rheumatology Unit, 2nd Department of Medicine and Laboratory, National and Kapodistrian University of Athens School of Medicine, General Hospital of Athens Hippokration, Athens, Greece; Nephrology Department, General Hospital of Athens Hippokration, Athens, Greece; Joint Rheumatology Program, Clinical Immunology-Rheumatology Unit, 4th Department of Medicine, National and Kapodistrian University of Athens School of Medicine, Attikon General Hospital, Athens, Greece; Joint Rheumatology Program, Clinical Immunology-Rheumatology Unit, 2nd Department of Medicine and Laboratory, National and Kapodistrian University of Athens School of Medicine, General Hospital of Athens Hippokration, Athens, Greece

**Keywords:** ANCA, patient reported outcomes, quality of life, vasculitis, rheumatoid arthritis

## Abstract

**Objectives:**

There are limited data regarding health-related quality of life (HRQoL) in patients with ANCA-associated vasculitides (AAVs). We aimed to evaluate the HRQoL in patients with AAVs and compare it to another chronic inflammatory disease like RA and to healthy controls (HC).

**Methods:**

This was a multicentre, cross-sectional study of patients with AAVs and RA recruited from three tertiary rheumatology clinics. HRQoL was assessed with the Short Form 36 Health Survey, which included the physical and mental component summary scores (PCS and MCS). Data from 1007 HC served as historical controls.

**Results:**

Sixty-six patients with AAVs and 71 with RA were included. Both AAV and RA patients had significantly lower PCS and MCS scores compared with HC (*P* < 0.05). HRQoL in AAV patients was worse in patients with microscopic polyangiitis compared with granulomatosis with polyangiitis (physical components) and those with high (VDI ≥ 3) *vs* low (VDI < 3) damage scores while it did not differ between those with active (BVASv3 ≥ 1) *vs.* inactive (BVASv3 < 1) disease. In contrast, in RA patients, HRQoL correlated both with disease activity (assessed by the DAS28-ESR) and functional impairment/damage (assessed by the HAQ). Although overall patients with RA had similar HRQoL compared with those with AAVs, those with active RA had worse HRQoL compared with those with active AAV.

**Conclusions:**

In patients with AAVs, HRQoL correlated more with organ damage and less with disease activity whereas in RA patients, it correlated with both. These data emphasize the need for AAV therapies aiming at preventing organ damage and thus improving HRQoL.

Rheumatology key messages

- Overall HRQoL was equally impaired in AAV and RA patients in comparison to healthy controls.
- In AAV patients, HRQoL correlated with organ damage whereas in RA it correlated additionally with disease activity.
- These data underpin the unmet therapeutic need for early damage prevention in AAV patients.

## Introduction

ANCA-associated vasculitides (AAVs) including granulomatosis with polyangiitis (GPA), microscopic polyangiitis (MPA) and eosinophilic granulomatosis with polyangiitis (EGPA) [1] are life-threatening diseases with significant morbidity and mortality. Despite significant therapeutic advances, many patients continue to experience refractory or relapsing disease. One-third of patients have irreversible damage at diagnosis, and over time many develop chronic complications related either to the disease itself or to the adverse effects of immuno-suppressive/modulatory therapies [2].

Health-related quality of life (HRQoL) in patients with AVVs has been found to be consistently affected in a number of studies using the Short Form 36 Health Survey (SF-36) [3–17]. While in some, SF-36 correlated with disease activity [3, 6, 12], in others, including a meta-analysis by Walsh *et al.* [10] no such correlation was observed [5, 7, 9, 13]. Although successful induction or maintenance treatment which decreases or prevents disease flares (by decreasing disease activity) could improve HRQoL, this has not been proven in long-term studies yet. Considering other chronic autoimmune inflammatory rheumatic diseases (AIRDs), it has been suggested that other factors may contribute to impaired HRQoL including fatigue, mood disorders, sleep disorders and inability to work [18]. Fatigue has been shown to be common in patients with AVVs, even in remission [19]. Impaired HRQoL is common also in other chronic inflammatory diseases such as RA [20–24].

The inflammatory burden of AAVs and the effect of organ damage associated both with the disease and its treatment has significant impact in the physical and mental components of the lives of patients. Whether this is comparable to the impact of other AIRDs remains unanswered. Until today, very few studies have investigated the comparative HRQoL of patients with AAVs to other chronic AIRDs like RA [4, 17]. The aim of our study was to assess the HRQoL in patients with AAVs and compare both to that of the general population and to that of patients with RA.

## Methods

### Study patients

We conducted a cross-sectional study of 137 patients with AAVs (*n* = 66) or RA (*n* = 71), who were actively followed in three referral, tertiary rheumatology centres in Athens, Greece. In addition, literature data from 1007 healthy controls (HC) from the same country served as historic controls [25]. All enrolled patients were adults (>18 years) who fulfilled the 2010 American College of Rheumatology (ACR)/European League Against Rheumatism (EULAR) criteria for RA [26] and the 2012 Chapel Hill Consensus Definitions for AAVs [1]. The study was approved by the Institutional Review Board (57/26–3-2018) and all patients signed an informed consent form. This study complies with the Declaration of Helsinki.

### Indices of disease activity, functional status/damage and HRQoL

For patients with AAVs, disease activity and damage were recorded in each assessment, using the Birmingham Vasculitis Activity Score version 3 (BVASv3) [27] and the Vasculitis Damage Index (VDI) [28], respectively. For RA patients, disease activity and functional impairment or damage were recorded using the Disease Activity Score in 28 joints (DAS28-ESR) [29] and the Health Assessment Questionnaire (HAQ) [30], respectively. Based on the BVASv3 and DAS28-ESR scores, AAV and RA patients were classified as those with ‘active’ (BVASv3 ≥ 1 or DAS28-ESR ≥ 3.2, respectively) or ‘inactive/low activity’ (BVASv3 < 1 or DAS28-ESR < 3.2, respectively) disease while based on the VDI and HAQ scores, AAV and RA patients were classified as those with ‘high’ (VDI ≥ 3 or HAQ ≥ 0.75 respectively) or ‘low’ (VDI < 3 or HAQ ≤ 0.63 respectively) damage scores. A VDI score of ≥5 has been proposed as a cut-off for increased damage [31], due to previously established links with mortality [32]. In our study, we used a lower cut-off of 3 in order to evaluate the effect of even ‘lower’ damage in HRQoL.

HRQoL was assessed with the SF-36 [33]. SF-36 is a generic measure of HRQoL that includes eight domains: physical function (PF), role limitations due to physical problems (RP), bodily pain (BP), general health perceptions (GH), vitality (VT), social function (SF), role limitations due to emotional problems (RE), and mental health (MH). These eight domains are summarized in the physical component (PCS) and mental component (MCS) summary scores. Each SF-36 domain is scored from 0 to 100, with higher scores representing better health. In 2005, the SF-36 was validated in the Greek language [25]. We used a scoring calculator for the evaluation of SF-36 questionnaire, available from the Orthotoolkit website (ttps://orthotoolkit.com/sf-36/). The method of calculating PCS and MCS is shown in Supplementary Data S1, available at *Rheumatology* online.

### Statistical methods

Dichotomous variables are shown as percentages while continuous variables are shown as mean (standard deviation) for nonparametric distributions, respectively. χ^2^ was used for comparison of dichotomous and Mann–Whitney or *t* test for continuous variables. For continuous variables we also used Pearson’s correlation coefficient. Statistical significance was considered for *P* values <0.05. GraphPad Prism 5.00 (GraphPad Software, Inc., San Diego, CA, USA) and SPSS 24.0 (SPSS software, Armonk, NY, USA) were used.

## Results

### Patient characteristics

A total of 137 patients with AAVs (*n* = 66) or RA (*n* = 71) were enrolled in the study. The majority of the AAVs patients had GPA (62%) and generalized disease (74%). Fifty-six percent of AAV patients were females, with a mean age of 63.4 years (Table 1) while the mean disease duration was 6 years. At the time of enrolment, 27% of patients had active disease (BVASv3 ≥ 1) and 79% demonstrated high damage scores (VDI ≥ 3). Among RA patients (*n* = 71), a similar proportion had active (DAS28-ESR ≥ 3.2) and more functionally impairing/damaging (HAQ ≥ 0.75) disease, 28% and 70% respectively. There were no significant differences in patient and disease characteristics between the two patient groups (Table 1). With regard to comorbidities, patients with AAV had higher frequencies of stroke, serious infections and end-stage renal disease (ESRD) compared with patients with RA (Supplementary Table S1, available at *Rheumatology* online).

**Table 1.** Patient and disease characteristics

	AAVs	RA	*P*
	(*n* = 66)	(*n* = 71)	
Female, *n* (%)	37 (56.1)	40 (56.3)	0.974
Age, years, mean (SD)	63.4 (15.7)	63.3 (13)	0.977
Disease duration, years, mean (SD)	6 (5)	6 (6.2)	0.928
AAV, *n* (%)			
GPA	41 (62)	NA	NA
MPA	19 (29)		
EGPA	6 (9)		
Severity, *n* (%)			
Generalized	49 (74)	NA	NA
Localized	17 (26)		
Activity, *n* (%)			0.907
Active disease	18 (27)	20 (28.2)	
Remission	48 (73)	51 (71.8)	
Damage/Functional impairment, *n* (%)			
High	52 (79)	50 (70)	0.262
Low	14 (21)	21 (30)	
Induction AAV therapies, *n* (%)			
CYC	29 (43.9)	—	
RTX	23 (34.8)	—	
RTX+CYC	6 (9.1)	—	
MTX	5 (7.6)	—	
GCs alone	3 (4.6)	—	
Current AAV therapies, *n* (%)			
RTX	44 (66.7)	—	
AZA	7 (10.6)	—	
MMF	5 (7.6)	—	
MTX	4 (6.1)	—	
GCs	28 (42.9)		
Mean (s.d.) daily dose, mg	4.7 (3.1)		
None	6 (9)	—	
Current RA therapies, *n* (%)			
csDMARDs	—	56 (78.9)	
bDMARDs	—	29 (40.8)	
tsDMARDs	—	5 (7)	
GCs	—	12 (16.9)	

The main characteristics of patients with AAVs and RA are shown and compared.

AAVs: ANCA-associated vasculitides; GPA: granulomatosis with polyangiitis; MPA: microscopic polyangiitis; RTX: rituximab; GCs: glucocorticoids; csDMARDs: conventional synthetic DMARDs; bDMARDs: biologic DMARDs; tsDMARDs: targeted synthetic DMARDs; NA: not applicable.

Patients with AAVs had received induction therapies either with cyclophosphamide (CYC, 43.9%), rituximab (RTX, 34.8%) or their combination (9.1%), in addition to glucocorticoids (GCs, Table 1). As maintenance therapies they received RTX (66.7%), azathioprine (AZA, 10.6%), mycopenolate mofetil (MMF, 7.6%) or methotrexate (MTX, 6.1%). At the time of evaluation, 53 patients were on maintenance therapies (80%), 4 on induction schemes (6%) while 6 patients (9%) were not on any treatment (Table 1). Among them, 42.4% were receiving GCs [mean prednisolone dose 4.7 mg/day (Table 1)].

The corresponding treatments for RA patients at the time of evaluation are also shown in Table 1.

### HRQoL of patients

#### Patients with AAVs or RA compared with HC

**Figure 1.**
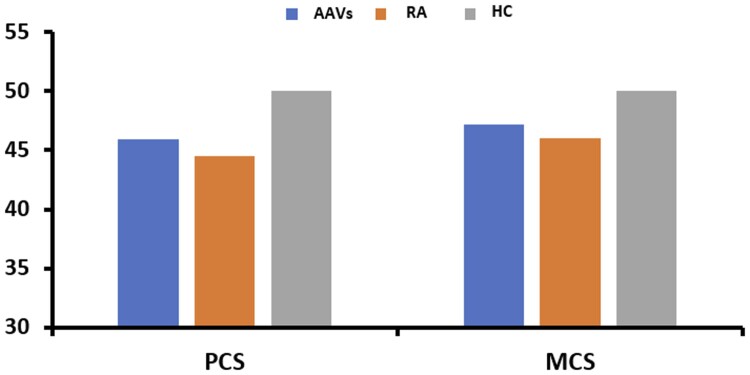
Comparison of physical and mental summary scales of health-related quality of life between patients with AAVs or RA and HC. The mean values of the PCS and MCS scores in each group are depicted. *P*-values for: PCS: physical component summary, AAVs *vs.* HC: *P* < 0.001, RA *vs.* HC: *P* < 0.001, AAVs *vs.* RA: *P* = 0.462. MCS: mental component summary, AAVs *vs.* HC: *P* = 0.024, RA *vs.* HC: MCS *P* = 0.001, AAVs *vs.* RA: *P* = 0.431. AAVs: ANCA-associated vasculitides; HC: healthy controls; RA: rheumatoid arthritis

Both groups of patients with AAVs and RA had overall significantly impaired HRQoL compared with HC as assessed globally by the PCS and MCS scores (*P* < 0.05, Fig. 1, Table 2). For the different SF-36 domains, for AAV patients, this was the case for physical function, role physical and mental health, while for RA patients for all domains except for vitality and social function (Table 2).

**Table 2.** Health-related quality of life of patients with AAVs or RA compared with HC

AAVs (*n* = 66)									
SF-36 summary scores and domains	AAVs—HC			RA—HC			C. Disease type		
	AAVs (*n* = 66)	HC (*n* = 1007)	*P*	RA (*n* = 71)	HC (*n* = 1007)	*P*	AAVs (*n* = 66)	RA (*n* = 71)	*P*
Physical component summary (PCS)	**45.9 (11.9)**	**50 (10)**	**<0.001**	**44.5 (10.3)**	**50 (10)**	**<0.001**	45.9 (11.9)	44.5 (10.3)	0.462
Mental component summary (MCS)	**47.2 (9.1)**	**50 (10)**	**0.024**	**46 (8.7)**	**50 (10)**	**0.001**	47.2 (9.1)	46 (8.7)	0.431
Physical function (PF)	**63.1 (30)**	**80.8 (25.6)**	**<0.001**	**63.6 (25.6)**	**80.8 (25.6)**	**<0.001**	63.1 (30)	63.6 (25.6)	0.916
Role physical (RP)	**67.7 (38.8)**	**79.7 (37.7)**	**0.012**	**58.6 (38.7)**	**79.7 (37.7)**	**<0.001**	67.7 (38.8)	58.6 (38.7)	0.171
Bodily pain (BP)	75.4 (15.2)	73 (31.7)	0.541	**59.5 (28.3)**	**73 (31.7)**	**<0.001**	**75.4 (15.2)**	**59.5 (28.3)**	**<0.001**
General health (GH)	63.9 (21.6)	67.5 (23.5)	0.226	**58.7 (19.8)**	**67.5 (23.5)**	**0.003**	63.9 (21.6)	58.7 (19.8)	0.143
Vitality (VT)	71.3 (22)	66.5 (22.4)	0.091	69.4 (19.5)	66.5 (22.4)	0.304	71.3 (22)	69.4 (19.5)	0.593
Social function (SF)	76.7 (26.1)	82.1 (28.1)	0.129	76.4 (21.2)	82.1 (28.1)	0.106	76.7 (26.1)	76.4 (21.2)	0.941
Role emotional (RE)	76.3 (29)	81.5 (36.3)	0.254	**63.1 (30.3)**	**81.5 (36.3)**	**<0.001**	**76.3 (29)**	**63.1 (30.3)**	**0.010**
Mental health (MH)	**55 (22.3)**	**68.2 (21.3)**	**<0.001**	**56.1 (21.4)**	**68.2 (21.3)**	**<0.001**	55 (22.3)	56.1 (21.4)	0.768

The comparisons in the physical and mental component summary scores as well as the individual components between the three groups (AAV, RA and HC) are shown.

Values are shown as mean (±1 standard deviation). The *P*-values in the comparisons between the different groups are shown.

Those with *P* < 0.05 are shown in bold.

SF-36: Short Form 36 Health Survey; AAVs: ANCA-associated vasculitides; RA: rheumatoid arthritis; HC: healthy controls.

#### Patients with AAVs

**Figure 2.**
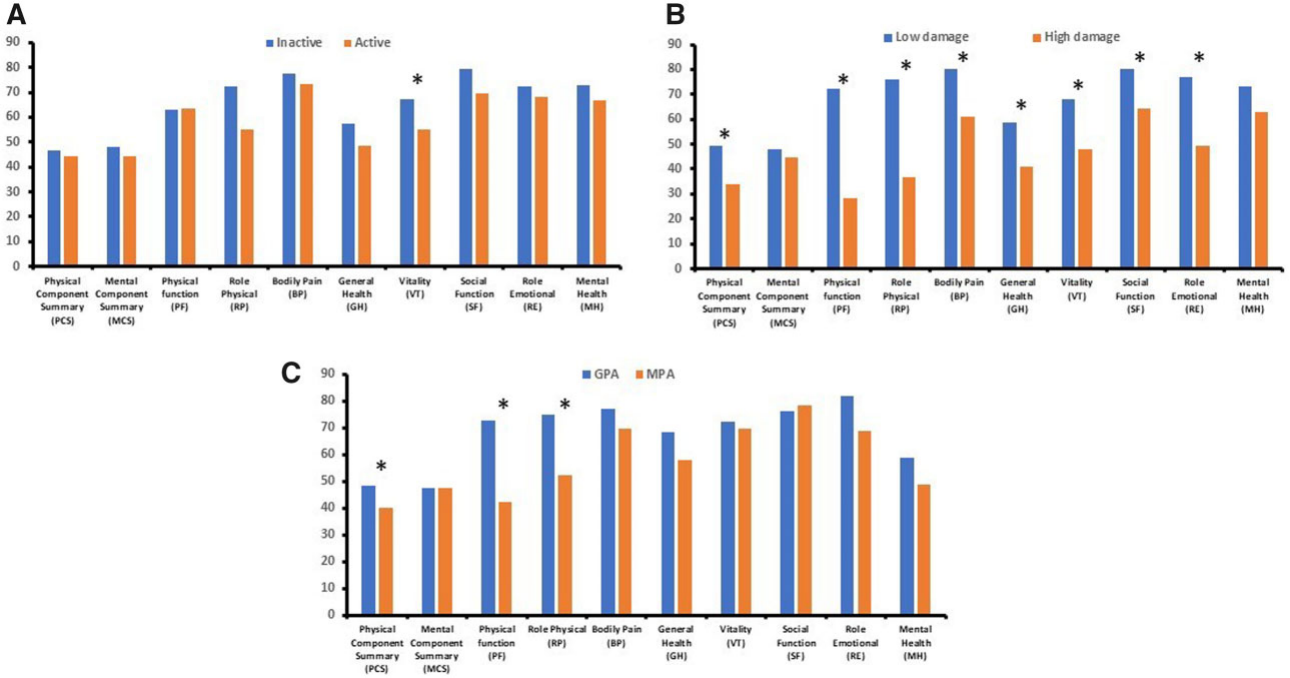
Health-related quality of life (HRQoL) in patients with AAV according to their disease: (**A**) **Activity** (inactive, BVASv3<3 *vs.* active, BVASv3≥3), (**B**) **Damage status** (low damage, VDI<3 *vs.* high damage, VDI≥3) and (**C**) **Type** (GPA *vs.* MPA). The mean scores of the summary and individual SF-36 scores in patients with AAVs according to their activity, damage status and clinical phenotype are shown. *P < 0.05. AAVs: ANCA-associated vasculitis; BVASv3: Birmingham Vasculitis Activity Score version 3; VDI: Vasculitis Damage Index; GPA: granulomatosis with polyangiitis; MPA: microscopic polyangiitis

The HRQoL in patients with AAVs was assessed in different patient subgroups according to their disease activity, organ damage status and type of AAV (Fig. 2, Supplementary Table S2 and S3, available at *Rheumatology* online).

We did not observe any correlation between disease activity (as assessed by the BVASv3 score) and HRQoL (except for general health, *P* = 0.012, Supplementary Table 2A, available at *Rheumatology* online). Similar results were obtained when the HRQoL was compared between those with active (BVASv3 ≥ 1) and inactive (BVASv3 < 1) disease (except for vitality, *P* = 0.038, Fig. 2A, Supplementary Table S3A, available at *Rheumatology* online).

On the contrary, HRQoL correlated with disease damage (as assessed by the VDI). This was the case for PCS (*P* < 0.0001) and most SF-36 domains (*P* < 0.05, except for bodily pain and vitality, Supplementary Table S1, available at *Rheumatology* online). When the comparative analysis was performed between those with high (VDI ≥3) *vs.* low (VDI < 3) damage scores, similar statistically significant differences were seen except for MCS and mental health (Fig. 2B and Supplementary Table S3B, available at *Rheumatology* online). No differences in HRQoL were seen according to disease duration or type of induction or maintenance therapies (data not shown).

Possible differences of HRQoL between patients with GPA (*n* = 41) and MPA (*n* = 19) were also explored. Patients with MPA appeared to have worse HRQoL compared with GPA as assessed by the PCS score (39.9 ± 12.5 *vs.* 48.3 ± 10.4, *P* = 0.009) and the domains of physical function and role physical (Fig. 2C and Supplementary Table S3C, available at *Rheumatology* online). Although there were no differences in comorbidities between the two groups (with the exception of chronic obstructive pulmonary disease, Supplementary Table S4, available at *Rheumatology* online), MPA patients were older, had more frequently generalized disease, worse kidney function and more damage compared with GPA patients (Supplementary Table S4, available at *Rheumatology* online) that could explain their decreased physical function scores.

#### Patients with RA

**Figure 3.**
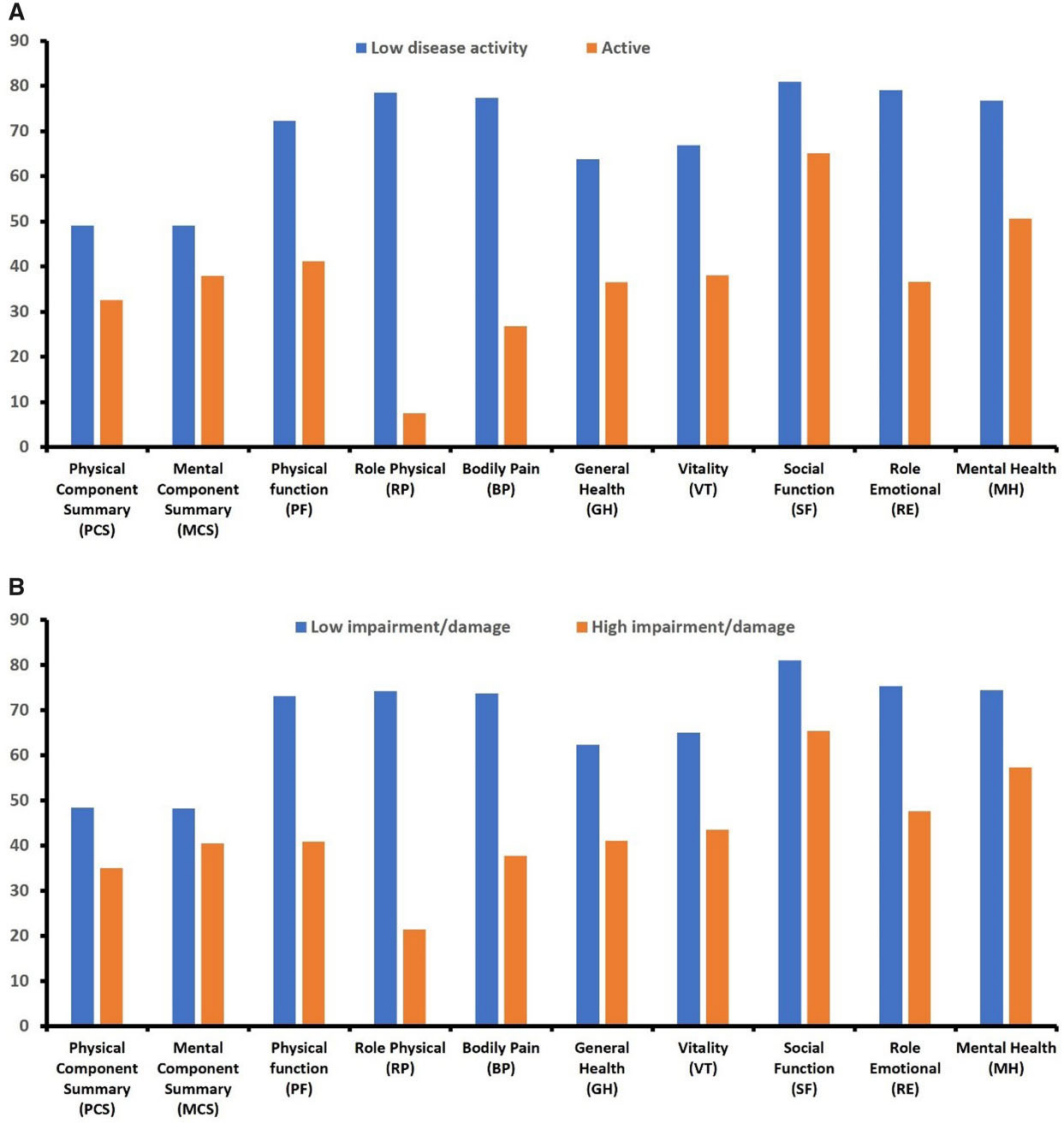
Health-related quality of life (HRQoL) in patients with RA according to their disease: (**A**) **Activity** (low disease activity, DAS28-ESR<3.2 *vs.* active disease, DAS28-ESR≥3.2). (**B**) **Damage status** (low functional impairment/damage HAQ≤0.63 *vs.* high functional impairment/damage HAQ≥0.75). The mean scores of the summary and individual SF-36 scores in patients with RA according to their disease activity and damage status are shown. For all comparisons *P* < 0.005. DAS28-ESR: Disease Activity Score in 28 joints

In RA patients HRQoL correlated both with disease activity (assessed by DAS28-ESR, *P* < 0.0001, Supplementary Table S2B, available at *Rheumatology* online) and functional impairment/damage (assessed by HAQ, *P* < 0.0001, Supplementary Table 2B, available at *Rheumatology* online). Similar results were seen when HRQoL indices were compared between those with active (DAS28-ESR ≥ 3.2) *vs.* low activity (DAS28-ESR < 3.2) disease status (Fig. 3A and Supplementary Table S5A, available at *Rheumatology* online) and those with high (HAQ ≥ 0.75) or low (HAQ < 0.63) functional impairment/damage scores (Fig. 3B and Supplementary Table S5B, available at *Rheumatology* online). We did not observe any differences in HRQoL among patients treated with different treatment regimens (csDMARDs, bDMARDs, tsDMARDs or their combinations, data not shown).

#### Patients with AAVs compared with RA

Overall, there was no statistically significant difference in the HRQoL between patients with AAVs and RA as assessed by the PCS and MCS scores (Table 2). This was also the case for all SF-36 domains except for bodily pain (worse in RA, 59.5 ± 28.3 *vs.* 75.4 ± 15.2, *P* < 0.001, Table 2) and role emotional (worse in RA, 63.1 ± 30.3 *vs.* 76.3 ± 29, *P* = 0.01, Table 2).

Nevertheless, when specific AAV subgroups according to their disease activity, damage status and disease type were examined and compared with RA, certain differences appeared.

According to disease activity, there were no difference in the HRQoL among patients with AAVs and RA who had inactive disease (Supplementary Table S6, available at *Rheumatology* online). In contrast, patients with active RA reported worse HRQoL in all areas, including PCS and MCS, except for social function (*P* = 0.6) and general health (*P* = 0.054), compared with patients with active AAV (Supplementary Table S6, available at *Rheumatology* online).

When patients from each disease were grouped according to their disease damage/functional impairment status, no significant differences were found except for bodily pain which was lower in RA patients with significant functional impairment/damage (HAQ ≥ 0.75) compared with AAV patients with high damage scores (VDI ≥ 3, *P* = 0.024, Supplementary Table S7, available at *Rheumatology* online).

When the HRQoL was assessed and compared according to the AAV type (GPA or MPA), it appeared that MPA and RA patients had a similar HRQoL except for bodily pain which was worse in RA (*P* = 0.047) and physical function which was worse in MPA patients (42.3 ± 30.3 *vs.* 63.5, *P* = 0.003, Supplementary Table S8, available at *Rheumatology* online). On the contrary, RA patients had lower scores compared with GPA patients in more components (PCS) and domains (role physical, bodily pain, general health, role emotional: Supplementary Table S8, available at *Rheumatology* online).

## Discussion

This is one of the few studies in the literature evaluating the HRQoL not only in patients with AAVs but also comparatively to patients with another chronic inflammatory disease like RA. Our study confirmed previous findings that patients with AAVs had worse HRQoL compared with the general population, which was similar to that of RA. Moreover, we found that the HRQoL was worse in those with MPA (than GPA) and correlated more with chronic damage than disease activity. That differed from RA where HRQoL correlated both with disease activity and functional impairment (indicative of chronic joint damage). The more specific tools assessing HRQoL in AAV patients such as AAV PRO may provide further insights in possible explanations for the observations of this study.

As shown in previous studies, both AAV [3–17] and RA [20–24] patients displayed worse HRQoL (measured by the PCS and MCS) compared with HC. This is most likely due to the direct and indirect effects of acute and chronic inflammation and organ damage caused by these two inflammatory diseases. The evaluation of HRQoL in patients with AAVs is complex due to its natural course with frequent relapses and long periods of therapy-induced remission, its clinical heterogeneity, and its multisystem involvement. In our study, the HRQoL in AAV patients was worse compared with HC, both in the physical and mental components. Herlyn *et al.*, in a study of 264 patients with vasculitis, reported that fatigue and reduced energy level were considered the most important disease burdens, compared with factors such as haemodialysis or oxygen dependence [34]. Impaired vitality in these patients correlated with biopsychosocial factors such as sleep disturbance and dysfunctional coping styles, as well as with disease activity (as assessed by CRP levels) [35].

In our study, HRQoL did not correlate with disease activity in AAV patients as measured by the SF-36. This could be due to the reduced sensitivity of the generic patient-reported outcome (PROs) questionnaires to assess disease-specific impairments that are important to patients [36]. The recently introduced AAV-specific PRO questionnaire (AAV-PRO) could be a more sensitive way to assess the HRQoL in these patients [36].

On the contrary, HRQoL correlated with all-cause damage as assessed by the VDI. Our findings are consistent with previous studies [37]. It could be that the damage seen is partially the result of suboptimal past therapies, which underlines the unmet need for more efficacious therapies able to prevent chronic damage inflicted either by the disease itself or the therapies used (especially GCs). Therapeutic developments in the field of AAVs with the advent of monoclonal biological therapies (RTX) have transformed the natural course of the disease. In terms of HRQoL, RTX displayed similar short-term improvements in SF-36 PCS and MCS compared with CYC when it was used as induction therapy [38]. On the other hand, when used as maintenance therapy it showed better improvement in PCS compared with AZA, potentially owing to its lower relapse rate [39, 40]. Whether or not newer therapies such as the recently approved C5a receptor inhibitor (Avacopan) [41] could have a better long-term effect on HRQoL by reducing GC-exposure is unclear.

One of the novel findings of our study was that MPA patients had significantly lower PCS score, indicating worse physical function compared with GPA patients. Although disease activity was similar between the two groups, MPA patients displayed more damage, were older and had worse renal function that could explain their impaired physical function. To our knowledge there is no other comparative study of AAV phenotypes and HRQoL in the literature.

Despite the disadvantages of generic PROs, such as SF-36, they can be used to compare HRQoL between patients with different chronic conditions. In our study, we chose RA as a chronic inflammatory disease to serve as a comparator. The compared populations had overall similar comorbidity burden with the exception of stroke, serious infections and ESRD, a finding potentially explained by the nature of each disease and the treatments used. RA patients demonstrated impaired HRQoL compared with HC that correlated both with disease activity (DAS28 score) and functional impairment indicative of joint damage (HAQ score). Our findings are in agreement with previous studies [20–24]. In a systemic review and meta-analysis of 22.335 patients with RA it was shown that they had worse HRQoL compared with HC, especially on the physical HRQoL components [24], while disease activity and functional status correlated with HRQoL [21].

When the HRQoL was compared between patients with AAVs and RA, overall, no significant differences were seen (except for bodily pain and role emotional). RA patients had a significantly lower bodily pain score (reflecting worse pain) compared with AAV patients regardless of their activity or damage status. These findings are in agreement with a study by Hinojosa-Azaola *et al.* [4] and can be explained by the significant effect of pain on the HRQoL of patients with RA [42], while patients with AAVs usually have milder joint involvement.

On the other hand, it is interesting that patients with active RA had more impaired HRQoL compared with patients with active AAV whereas this was not the case for the other patient subgroups (those with inactive, low or high damage disease). This, as mentioned above, could be due to the specific disease characteristics or to the inability of SF-36 to capture specifically the HRQoL impairment in patients with active AAVs.

We acknowledge that our study has certain limitations. Firstly, its cross-sectional design could have excluded newly diagnosed and included more prevalent cases. However, this design provides a more representative image of a real-life AAV cohort and also enables us to appreciate the effect of AAV-related damage to HRQoL. Secondly, we utilized a previous large database of HC instead of newly recruited HC. Finally, we did not use the AAV-specific questionnaire (AAV-PRO) to assess HRQoL, but the generic nature of SF-36 enabled us to make comparisons with another AIRD such as RA.

## Conclusion

Our study is one the few comparative studies in the literature showing that patients with AAVs have impaired HRQoL, similar to RA, which was more prominent in MPA patients and those with high organ damage scores. The more specific tools assessing HRQoL in AAV patients such as AAV PRO need to be validated in larger patient populations whereas newer treatment strategies aiming to prevent organ damage in AAVs are urgently needed.

## Supplementary Material

kead214_Supplementary_Data

## Data Availability

The datasets of the study are available from the corresponding author on reasonable request.
