# Supplementary material for: Health-related quality of life in patients with ANCA vasculitides compared to rheumatoid arthritis: a cross-sectional comparative study
Source: Rheumatology (Oxford). 2023 May 12;63(2):399–406. doi: 10.1093/rheumatology/kead214 (PMC10836997; doi:10.1093/rheumatology/kead214)
Supplement: kead214_Supplementary_Data [file kead214_supplementary_data.docx]

**Supplementary Tables**

**Suppl. Table S1**

**Comorbidities in AAVs and RA patients**

|  | **AAVs**  (n= 66) | **RA**  (n=71) | ***p*** |
| --- | --- | --- | --- |
| **COPD** | 5 (7.6) | 4 (5.6) | 0.648 |
| **CVD** | 12 (18.2) | 10 (14.1) | 0.516 |
| **Stroke** | 5 (7.6) | 0 (0) | **0.019** |
| **Hypertension** | 26 (39.4) | 30 (42.3) | 0.735 |
| **Depression** | 12 (18.2) | 10 (14.1) | 0.516 |
| **Diabetes** | 8 (12.1) | 14 (19.7) | 0.228 |
| **Serious Infection** | 13 (19.7) | 5 (7) | **0.013** |
| **ESRD** | 3 (4.5) | - | **0.070** |
| **Cancer** | 7 (10.6) | 4 (5.6) | 0.463 |

p<0.05 for comparisons are shown in bold in grey boxes

RA: Rheumatoid arthritis, AAVs: ANCA-associated vasculitis, COPD: chronic obstructive pulmonary disease, CVD: cardiovascular disease (myocardial infarction, coronary artery bypass graft, percutaneous coronary intervention), ESRD: end stage renal disease

**Suppl. Table S2**

**Correlation between health-related quality of life (SF-36) and indices of disease activity and damage in patients with A. AAVs and B. RA**

| **SF-36 Summary Scores**  **and Domains** | 1. **AAVs** | | | | 1. **RA** | | | |
| --- | --- | --- | --- | --- | --- | --- | --- | --- |
|  | **Disease activity**  **(BVASv3)** | | **Disease damage**  **(VDI)** | | **Disease activity**  **(DAS28-ESR)** | | **Disease damage**  **(HAQ)** | |
|  | **Pearson Correlation** | **p** | **Pearson Correlation** | **p** | **Pearson Correlation** | **p** | **Pearson Correlation** | **p** |
| **Physical Component Summary (PCS)** | -0.007 | 0.955 | **-0.503** | **<0.0001** | -0.720 | **<0.0001** | -0.691 | **<0.0001** |
| **Mental Component Summary (MCS)** | -0.161 | 0.198 | -0.182 | 0.143 | -0.542 | **<0.0001** | -0.586 | **<0.0001** |
| **Physical function (PF)** | 0.049 | 0.696 | **-0.592** | **<0.0001** | -0.614 | **<0.0001** | -0.668 | **<0.0001** |
| **Role Physical (RP)** | -0.058 | 0.643 | **-0.390** | **<0.0001** | -0.739 | **<0.0001** | -0.696 | **<0.0001** |
| **Bodily Pain (BP)** | 0.088 | 0.483 | 0.003 | 0.981 | -0.499 | **<0.0001** | -0.446 | **<0.0001** |
| **General Health (GH)** | **-0.306** | **0.012** | **-0.359** | **0.003** | -0.680 | **<0.0001** | -0.638 | **<0.0001** |
| **Vitality (VT)** | -0.146 | 0.243 | -0.219 | 0.077 | -0.567 | **<0.0001** | -0.577 | **<0.0001** |
| **Social Function (SF)** | -0.093 | 0.460 | **-0.282** | **0.022** | -0.383 | **<0.0001** | -0.503 | **<0.0001** |
| **Role Emotional (RE)** | -0.058 | 0.641 | **-0.266** | **0.031** | -0.739 | **<0.0001** | -0.669 | **<0.0001** |
| **Mental Health (MH)** | -0.072 | 0.567 | **-0.343** | **0.005** | -0.518 | **<0.0001** | -0.576 | **<0.0001** |

p<0.05 for comparisons are shown in bold in grey boxes.

SF-36: Short Form 36 Health Survey, AAVs: ANCA-associated vasculitis, RA: Rheumatoid arthritis, BVASv3: Birmingham Vasculitis Activity Score version 3., VDI: Vasculitis Damage Index, DAS28-ESR: Disease Activity Score in 28 joints, HAQ: Health Assessment Questionnaire

**Suppl. Table S3**

**Health-related quality of life between patients with AAVs according to their disease A. activity, B. damage status and C. type**

| **AAVs (n=66)** | | | | | | | | | | |
| --- | --- | --- | --- | --- | --- | --- | --- | --- | --- | --- |
| **SF-36 Summary Scores**  **and Domains** | **A. Disease activity status** | | | **B. Damage status** | | | **C. Disease type** | | | |
|  | **Inactive**  **BVASv3<1**  (n=48) | **Active**  **BVASv3≥1**  (n=18) | ***p*** | **Low damage**  **VDI<3**  (n=52) | **High damage**  **VDI≥3**  (n=14) | ***p*** | **GPA**  (n=41) | **MPA**  (n=19) | ***p*** |  |
| **Physical Component Summary (PCS)** | 46.5 (11.5) | 44.1 (13.0) | 0.451 | **49.1 (10.2)** | **34 (10.1)** | **<0.001** | **48.3 (10.4)** | **39.9 (12.5)** | **0.009** |  |
| **Mental Component Summary (MCS)** | 48.2 (9.1) | 44.3 (8.6) | 0.113 | 47.9 (8.7) | 44.7 (10.0) | 0.240 | 47.6 (9.1) | 47.5 (9.2) | 0.969 |  |
| **Physical function (PF)** | 62.9 (30.4) | 63.6 (29.7) | 0.934 | **72.4 (24.9)** | **28.5 (20.7)** | **<0.001** | **72.5 (26.1)** | **42.3 (30.3)** | **0.001** |  |
| **Role Physical (RP)** | 72.3 (37.6) | 55.0 (39.9) | 0.105 | **75.9 (34.2)** | **36.7 (39.9)** | **0.001** | **75.0 (33)** | **52.1 (43.3)** | **0.05** |  |
| **Bodily Pain (BP)** | 77.3 (28.4) | 73.4 (31.0) | 0.628 | **80.3 (27.7)** | **61.2 (29.5)** | **0.027** | 76.9 (14.9) | 69.8 (16.4) | 0.099 |  |
| **General Health (GH)** | 57.3 (23.7) | 48.6 (17.1) | 0.156 | **58.7 (21.7)** | **41.0 (19.2)** | **0.008** | 68.2 (19.9) | 57.8 (22.3) | 0.076 |  |
| **Vitality (VT)** | **67.2 (20.7)** | **55.0 (21.8)** | **0.038** | **68.2 (19.5)** | **47.8 (21.7)** | **0.001** | 72.0 (20.8) | 69.4 (21.7) | 0.662 |  |
| **Social Function (SF)** | 79.4 (24.5) | 69.4 (29.4) | 0.168 | **80.0 (23.6)** | **64.2 (31.7)** | **0.044** | 76.2 (26) | 78.2 (26.3) | 0.776 |  |
| **Role Emotional (RE)** | 72.2 (37.8) | 68.1 (37.0) | 0.697 | **76.9 (34.6)** | **49.5 (40.6)** | **0.014** | 81.9 (23.7) | 68.9 (34) | 0.145 |  |
| **Mental Health (MH)** | 73.0 (20.8) | 66.6 (24.8) | 0.297 | 73.4 (19.5) | 63.0 (28.8) | 0.118 | 58.6 (22.5) | 48.9 (21.5) | 0.121 |  |

The values are shown as mean (± 1 Standard Deviation)

p<0.05 for comparisons are shown in bold in grey boxes.

SF-36: Short Form 36 Health Survey, AAVs: ANCA-associated vasculitis, BVASv3: Birmingham Vasculitis Activity Score version 3, VDI: Vasculitis Damage Index, GPA: Granulomatosis with polyangiitis, MPA: Microscopic polyangiitis

**Suppl. Table S4**

**Differences between GPA and MPA patients**

|  | **GPA**  (n= 41) | **MPA**  (n=19) | ***p*** |
| --- | --- | --- | --- |
| **Age, years, mean (SD)** | 60.8 (16.4) | 71.7 (10.4) | **0.003** |
| **Disease duration, years, mean (SD)** | 5.9 (4.5) | 5.3 (5.5) | 0.716 |
| **Severity, n (%)**   - Generalized - Localized | 27 (65.8)  14 (34.2) | 18 (94.7)  1 (5.3) | **0.017** |
| **Activity, n (%)**   - Active disease - Remission | 11 (26.8)  30 (73.2) | 5 (26.3)  14 (73.7) | 0.967 |
| **Damage/Functional impairment, n (%)**   - High - Low | 4 (9.8)  37 (90.2) | 9 (47.4)  10 (52.6) | **0.001** |
| **Creatinine clearance at diagnosis, mean (SD)** | 75.1 (30.6) | 44.9 (29.5) | **0.001** |
| **Organ/System Involvement, n (%)** |  |  |  |
| Lung | 36 (87.8) | 17 (89.5) | 0.853 |
| Kidney | 22 (53.7) | 18 (94.7) | **0.002** |
| Creatinine Clearance below 30 ml/min | 5 (12.2) | 7 (36.8) | **0.028** |
| Nervous | 11 (26.8) | 2 (10.5) | 0.157 |
| Mucous/Eyes | 8 (19.5) | 1 (5.3) | 0.154 |
| ENT | 26 (63.4) | 5 (26.3) | **0.008** |
| Skin | 7 (17.1) | 2 (10.5) | 0.512 |
| Joints | 15 (36.6) | 10 (52.6) | 0.245 |
| **Comorbidities, n (%)** |  |  |  |
| COPD | 1 (2.4) | 4 (21.1) | **0.016** |
| CVD | 6 (14.6) | 6 (31.6) | 0.130 |
| Stroke | 2 (4.9) | 2 (10.5) | 0.418 |
| Hypertension | 16 (39) | 10 (52.6) | 0.327 |
| Depression | 6 (14.6) | 5 (26.3) | 0.281 |
| Diabetes | 6 (14.6) | 2 (10.5) | 0.666 |
| Current cancer | 7 (17.1) | 0 (0) | 0.057 |
| Serious infection | 8 (19.5) | 4 (21.1) | 0.891 |
| ESRD | 1 (2.4) | 2 (10.5) | 0.185 |

The values are shown as mean (± 1 Standard Deviation)

p<0.05 for comparisons are shown in bold in grey boxes.

GPA: Granulomatosis with polyangiitis, MPA: Microscopic polyangiitis, ENT: ear nose throat, COPD: chronic obstructive pulmonary disease, CVD: cardiovascular disease (myocardial infarction, coronary artery bypass graft, percutaneous coronary intervention), ESRD: end stage renal disease

**Suppl. Table S5**

**Health-related quality of life of patients with RA according to their disease A. activity and B. functional impairment/damage status**

| **RA (n=71)** | | | | | | |
| --- | --- | --- | --- | --- | --- | --- |
| **SF-36 Summary Scores**  **and Domains** | **A. Disease activity status** | | | **B. Functional impairment/damage status** | | |
|  | **Low disease activity**  **DAS28ESR<3.2**  (n=51) | **Active disease**  **DAS28ESR≥3.2**  (n=20) | ***p-value*** | **Low impairment/ damage**  **HAQ≤0.63**  (n=50) | **High**  **Impairment/ damage**  **HAQ≥0.75**  (n=21) | ***p-value*** |
| **Physical Component Summary (PCS)** | **49.1 (7.2)** | **32.6 (6.7)** | ***< 0.001*** | **48.4 (8.6)** | **35 (7.2)** | ***<0.001*** |
| **Mental Component Summary (MCS)** | **49.1 (7.1)** | **37.9 (7.0)** | ***< 0.001*** | **48.2 (7.6)** | **40.5 (8.6)** | ***<0.001*** |
| **Physical function (PF)** | **72.3 (22.7)** | **41.2 (18.3)** | ***< 0.001*** | **73.1 (22.4)** | **40.9 (17.2)** | ***< 0.001*** |
| **Role Physical (RP)** | **78.6 (23.8)** | **7.5 (14.2)** | ***< 0.001*** | **74.2 (31.8)** | **21.4 (26.5)** | ***< 0.001*** |
| **Bodily Pain (BP)** | **77.3 (19.6)** | **26.8 (21.1)** | ***< 0.001*** | **73.7 (24.2)** | **37.7 (28.6)** | ***< 0.001*** |
| **General Health (GH)** | **63.7 (16.7)** | **36.5 (20.0)** | ***< 0.001*** | **62.3 (18.5)** | **41.1 (20.9)** | ***< 0.001*** |
| **Vitality (VT)** | **66.9 (14.4)** | **38.0 (16.4)** | ***< 0.001*** | **65.1 (16.7)** | **43.5 (18.5)** | ***< 0.001*** |
| **Social Function (SF)** | **80.9 (19.3)** | **65.0 (22.0)** | ***0.004*** | **81.0 (19.1)** | **65.4 (22.3)** | ***0.004*** |
| **Role Emotional (RE)** | **79.1 (28.3)** | **36.6 (34.0)** | ***< 0.001*** | **75.3 (32.8)** | **47.6 (34.2)** | ***0.002*** |
| **Mental Health (MH)** | **76.7 (13.7)** | **50.6 (19.7)** | ***< 0.001*** | **74.4 (16.1)** | **57.3 (21.6)** | ***< 0.001*** |

The values are shown as mean (± 1 Standard Deviation)

p<0.05 for comparisons are shown in bold in grey boxes.

SF-36: Short Form 36 Health Survey, RA: Rheumatoid arthritis, DAS28-ESR: Disease Activity Score in 28 joints, HAQ: Health Assessment Questionnaire

**Suppl. Table S6**

**Comparison of health-related quality of life between patients with AAVs and RA according to their disease activity status**

| **SF-36 Summary Scores**  **and Domains** | **Inactive Disease** | | | | **Active Disease** | | | |
| --- | --- | --- | --- | --- | --- | --- | --- | --- |
|  | **AAVs**  (n=48) | **RA**  (n=51) | ***p*** | **AAVs**  (n=18) | | **RA**  (n=20) | ***p*** |  |
| **Physical Component Summary (PCS)** | 46.5 (11.5) | 49.1 (7.2) | 0.188 | **44.1 (13.0)** | | **32.6 (6.7)** | **0.001** |  |
| **Mental Component Summary (MCS)** | 48.2 (9.1) | 49.1 (7.1) | 0.611 | **44.3 (8.6)** | | **37.9 (7.0)** | **0.018** |  |
| **Physical function (PF)** | 62.9 (30.4) | 72.3 (22.7) | 0.082 | **63.6 (29.7)** | | **41.2 (18.3)** | **0.008** |  |
| **Role Physical (RP)** | 72.3 (37.6) | 78.6 (23.8) | 0.324 | **55.0 (39.9)** | | **7.5 (14.2)** | **<0.001** |  |
| **Bodily Pain (BP)** | 77.3 (28.4) | 77.3 (19.6) | 0.985 | **73.4 (31.0)** | | **26.8 (21.1)** | **<0.001** |  |
| **General Health (GH)** | 57.3 (23.7) | 63.7 (16.7) | 0.126 | 48.6 (17.1) | | 36.5 (20.0) | 0.054 |  |
| **Vitality (VT)** | 67.2 (20.7) | 66.9 (14.4) | 0.905 | **55.0 (21.8)** | | **38.0 (16.4)** | **0.01** |  |
| **Social Function (SF)** | 79.4 (24.5) | 80.9 (19.3) | 0.743 | 69.4 (29.4) | | 65.0 (22.0) | 0.600 |  |
| **Role Emotional (RE)** | 72.2 (37.8) | 79.1 (28.3) | 0.307 | **68.1 (37.0)** | | **36.6 (34.0)** | **0.01** |  |
| **Mental Health (MH)** | 73.0 (20.8) | 76.7 (13.7) | 0.293 | **66.6 (24.8)** | | **50.6 (19.7)** | **0.034** |  |

The values are shown as mean (± 1 Standard Deviation)

p<0.05 for comparisons are shown in bold in grey boxes.

SF-36: Short Form 36 Health Survey, AAVs: ANCA-associated vasculitis, RA: Rheumatoid arthritis

**Suppl. Table S7**

**Comparison of health-related quality of life between patients with AAVs and RA according to their disease damage status**

| **SF-36 Summary Scores**  **and Domains** |  | | | | | |
| --- | --- | --- | --- | --- | --- | --- |
|  | **Low damage/functional impairment** | | | **High damage/functional impairment** | | |
|  | **AAVs**  (n=52) | **RA**  (n=50) | **p** | **AAVs**  (n=14) | **RA**  (n=21) | **p** |
| **Physical Component Summary (PCS)** | 49.1 (10.2) | 48.4 (8.6) | 0.709 | 33.9 (10.1) | 35.0 (7.2) | 0.708 |
| **Mental Component Summary (MCS)** | 47.9 (8.7) | 48.2 (7.6) | 0.853 | 44.7 (10.0) | 40.5 (8.6) | 0.193 |
| **Physical function (PF)** | 72.4 (24.9) | 73.1 (22.4) | 0.862 | 28.5 (20.7) | 40.9 (17.2) | 0.062 |
| **Role Physical (RP)** | 75.9 (34.2) | 74.2 (31.8) | 0.711 | 36.7 (39.9) | 21.4 (26.5) | 0.181 |
| **Bodily Pain (BP)** | 80.3 (27.7) | 73.7 (24.2) | 0.203 | **61.2 (29.5)** | **37.7 (28.6)** | **0.024** |
| **General Health (GH)** | 58.7 (21.7) | 62.3 (18.5) | 0.370 | 41.0 (19.2) | 41.1 (20.9) | 0.988 |
| **Vitality (VT)** | 68.2 (19.5) | 65.1 (16.7) | 0.384 | 47.8 (21.7) | 43.5 (18.5) | 0.533 |
| **Social Function (SF)** | 80.0 (23.6) | 81.0 (19.1) | 0.814 | 64.2 (31.7) | 65.4 (22.3) | 0.896 |
| **Role Emotional (RE)** | 76.9 (34.6) | 75.3 (32.8) | 0.811 | 49.5 (40.6) | 47.6 (34.2) | 0.882 |
| **Mental Health (MH)** | 73.4 (19.5) | 74.4 (16.1) | 0.778 | 63.0 (28.8) | 57.3 (21.6) | 0.508 |

The values are shown as mean (± 1 Standard Deviation)

p<0.05 for comparisons are shown in bold in grey boxes.

SF-36: Short Form 36 Health Survey, AAVs: ANCA-associated vasculitis, RA: Rheumatoid arthritis

**Suppl. Table S8**

**Comparison of health-related quality of life between patients with GPA or MPA and RA**

| **SF-36 Summary Scores**  **and Domains** | **GPA vs. RA** | | | **MPA vs. RA** | | |
| --- | --- | --- | --- | --- | --- | --- |
|  | **GPA**  (n=41) | **RA**  (n=71) | ***p*** | **MPA**  (n=19) | **RA**  (n=71) | ***p*** |
| **Physical Component Summary (PCS)** | **48.3 (10.4)** | **44.4 (10.2)** | **0.05** | 39.9 (12.5) | 44.4 (10.2) | 0.107 |
| **Mental Component Summary (MCS)** | 47.6 (9.1) | 45.9 (8.6) | 0.341 | 47.5 (9.2) | 45.9 (8.6) | 0.492 |
| **Physical function (PF)** | 72.5 (26.1) | 63.5 (25.6) | 0.07 | **42.3 (30.3)** | **63.5 (25.6)** | **0.003** |
| **Role Physical (RP)** | **75.0 (33)** | **58.5 (38.7)** | **0.025** | 52.1 (43.3) | 58.5 (38.7) | 0.529 |
| **Bodily Pain (BP)** | **76.9 (14.9)** | **59.5 (28.2)** | **0.001** | **69.8 (16.4)** | **59.5 (28.2)** | **0.047** |
| **General Health (GH)** | **68.2 (19.9)** | **58.7 (19.7)** | **0.01** | 57.8 (22.3) | 58.7 (19.7) | 0.874 |
| **Vitality (VT)** | 72.0 (20.8) | 69.3 (19.4) | 0.503 | 69.4 (21.7) | 69.3 (19.4) | 0.992 |
| **Social Function (SF)** | 76.2 (26) | 76.4 (21.1) | 0.969 | 78.2 (26.3) | 76.4 (21.1) | 0.745 |
| **Role Emotional (RE)** | **81.9 (23.7)** | **63.0 (30.3)** | **0.001** | 68.9 (34) | 63.0 (30.3) | 0.469 |
| **Mental Health (MH)** | 58.6 (22.5) | 56.0 (21.4) | 0.545 | 48.9 (21.5) | 56.0 (21.4) | 0.203 |

The values are shown as mean (± 1 Standard Deviation)

p<0.05 for comparisons are shown in bold in grey boxes.

SF-36: Short Form 36 Health Survey, GPA: Granulomatosis with polyangiitis, MPA: Microscopic polyangiitis, RA: Rheumatoid arthritis

**Supplementary Data S1**

**Summary Scale Calculation Algorithm (SPSS syntax)**

*do if not missing (SF_1) and not missing (SF_2) and not missing (SF_3) and not missing (SF_4)

*and not missing (SF_5) and not missing (SF_6) and not missing (SF_7) and not missing (SF_8).

*compute SF_SP = (SF_1 * 0.504 + SF_2 * 0.384 + SF_3 * 0.279 + SF_4 * 0.328 + SF_5 * 0.017

* - SF_6 * 0.051 - SF_7 * 0.160 - SF_8 * 0.345+ 55.6) / 206.8 * 100.

*compute SF_SM = (-SF_1 * 0.298 - SF_2 * 0.176 - SF_3 * 0.079 - SF_4 * 0.107 + SF_5 * 0.236

* + SF_6 * 0.302 + SF_7 * 0.392 + SF_8 * 0.584+66)/217.4*100.

*end if.

*variable labels SF_SP 'Α. Physical Component Summary'.

*variable labels SF_SM 'Β. Mental Component Summary'.

execute.

do if not missing (SF_1).

compute PF_Z = (SF_1-84.52404)/22.89490.

end if.

do if not missing (SF_2).

compute RP_Z = (SF_2 - 81.19907)/33.79729.

end if.

do if not missing (SF_3).

compute BP_Z = (SF_3 - 75.49196)/23.55879.

end if.

do if not missing (SF_4).

compute GH_Z = (SF_4 - 72.21316)/20.16964.

end if.

do if not missing (SF_5).

compute VT_Z= (SF_5-61.05453)/20.86942.

end if.

do if not missing (SF_6).

compute SF_Z = (SF_6 - 83.59753)/22.37642.

end if.

do if not missing (SF_7).

compute RE_Z = (SF_7 - 81.29467)/33.02717.

end if.

do if not missing (SF_8).

compute MH_Z = (SF_8 - 74.84212)/18.01189.

end if.

execute.

do if NVALID (PF_Z, RP_Z, BP_Z, GH_Z, VT_Z, SF_Z, RE_Z, MH_Z) = 8.

compute AGG_PHYS= PF_Z * 0.42402 + RP_Z * 0.35119 + BP_Z * 0.31754

+ GH_Z * 0.24954 + VT_Z * 0.02877 - SF_Z * 0.00753 - RE_Z * 0.19206 - MH_Z

* 0.22069.

compute AGG_MENT= - PF_Z * 0.22999 - RP_Z * 0.12329 - BP_Z * 0.09731

- GH_Z * 0.01571 + VT_Z * 0.23534 + SF_Z * 0.26876 + RE_Z * 0.43407 + MH_Z

* 0.48581.

else.

compute AGG_PHYS=999.

compute AGG_MENT=999.

end if.

recode AGG_PHYS (999=SYSMIS).

recode AGG_MENT (999=SYSMIS).

do if not missing (AGG_PHYS).

compute SF_PCS = 50 + AGG_PHYS * 10.

end if.

do if not missing (AGG_MENT).

compute SF_MCS = 50 + AGG_MENT * 10.

end if.

variable labels SF_PCS 'Α. Physical Component Summary'.

variable labels SF_MCS 'Β. Mental Component Summary'.

execute.
